# Musk in Hiccough

**Published:** 1870-04

**Authors:** J. E. O’Brien

**Affiliations:** Phillipsburg, N. J.


ARTICLE III.—*Musk.* By J. E. O'BRIEN, M.D., Phillipsburg, N. J.

AMONG the specifics used to be considered musk for hiccough. While legitimate causes have happily removed many other drugs from the list, musk is in a fair way to succumb to the evil effects of adulteration.

I think it was Prof. Wood who, after trying several druggists' specimens, succeeded with a pod taken from his own cabinet. As far as I can learn from others, failure now attends every prescription of musk. In a recent search for the pure drug, one of two New York professors of *Materia Medica* upon whom I called informed me that he had ceased to lecture upon musk, so little confidence was felt in it there.

In this connection, I offer the following case, premising that the patient was a personal friend:

January 12. J. K. Erysipelas of face. Upon second day, congestion of the brain. The usual means: tinct. iodine, iron, bleeding, shaving of the head, ice to the scalp, enema, et al., were used in their appropriate places. Upon the ninth day delirium had ceased, pulse soft, skin moist, face still swollen.

January 21. Patient began to hiccough in morning; kept it up all day. I prescribed musk; Dr. Cavanaugh, of Easton, in consultation, prescribed blister. Morphia at night.

January 22. Notwithstanding morphia, hiccoughed all night, and still at it; Dr. C. thinks he will die; Dr. Field, of

Easton, called; recommends Hoffman's anodyne; also chloroform to epigastrium. Suggestions carried out.

Having exhausted the resources of this place in musk, Dr. F. obtains some in Easton. Given: grs. x every two hours.

23rd. I obtained a fresher article in Easton, at $32 per ℥; it is given from noon until ʒj has been taken.

24th. Hiccoughing steadily except an intermission of three hours, from 7 to 10 A. M., which we refer to the last prescription of musk. We now give quinia.

℞ Quinia Sulph., - - - - - - - - - ʒj;
Morphia Sulph., - - - - - - - - grs. ij;
M. Ft. Pulv. No. vj. S. One every hour.

This produced deafness, but the hiccough continued unabated, the spasms occurring every second, with exhausting force and painful regularity.

25th. No change; hiccoughing steadily; patient nearly worn out.

26th. Believing that pure musk, could it be obtained, would stop the hiccough and prevent death from exhaustion, I determined to seek the pure drug, and went to New York for the purpose of obtaining the assistance of Professors of Materia Medica in the search. The professors of two different colleges very kindly recommended me to a reliable Broadway druggist, who offered me powdered musk, but I was skeptical of powders, and refused. Finally, at the old drug house of W. H. Schieffelin & Co., I obtained two fine specimens of the glands from Moschus Moschiferres, containing about ʒ ij of powder each; price $18.40. Getting back at 7 P. M., find the patient rapidly sinking under the unremitting spasms of hiccough. I leave six powders of grs. xij each, directing one every two hours.

27th. At 5 A. M., when last powder was given, hiccough ceased. No more until 1 P. M., hiccough for twenty minutes, then ceased.

28th. At 5 A. M. "a few draws." This was the last of the hiccough. Gave quinia sulph, ʒ ij during the day.

Patient very sore about precordia; relieved by blister. All through the case patient took beef tea, made rich with cream, and in the last week, brandy. Some sequela occurred in the shape of cough, also an abscess over middle of right sterno mastoid, which discharged freely. The patient gradually gained strength, and now, February 20th, is able to walk about.

Among other remedies, I prescribed:

℞ Potass. Bromidi, - - - - - - - ℥ ss;
Tinct. Card. Comp., - - - - - - ℥ vj.
M. S. Tablespoonful three times daily.

But he failed to take it.

We are satisfied that pure musk saved this man.

